# Remineralization potential of fluoride and amorphous calcium phosphate-casein phospho peptide on enamel lesions: An *in vitro* comparative evaluation

**DOI:** 10.4103/0972-0707.62634

**Published:** 2010

**Authors:** S Lata, N O Varghese, Jolly Mary Varughese

**Affiliations:** Department of Conservative Dentistry and Endodontics, Thiruvananthapuram; 1 Government Dental College, Thiruvananthapuram

**Keywords:** ACP-CPP, demineralization-remineralization, fluoride

## Abstract

**Aim:**

This *in vitro* study was conducted on enamel blocks of human premolars with the aim of evaluating the remineralization potential of fluoride and ACP-CPP and the combination of ACP-CPP and fluoride on early enamel lesions.

**Materials and Methods:**

Fifteen intact carious free human premolars were selected. The coronal part of each tooth was sectioned into four parts to make 4 enamel blocks. The baseline SMH (surface microhardness) was measured for all the enamel specimens using Vickers microhardness (VHN) testing machine. Artificial enamel carious lesions were created by inserting the specimens in demineralization solution for 3 consecutive days. The SMH of the demineralised specimens was evaluated. Then the four enamel sections of each tooth were subjected to various surface treatments, i.e. Group 1- Fluoride varnish, Group 2- ACP-CPP cream, Group 3- Fluoride + ACP-CPP & Group 4- Control (No surface treatment). A caries progression test (pH cycling) was carried out, which consisted of alternative demineralization (3hours) and remineralization with artificial saliva (21 hours) for five consecutive days. After pH cycling again SMH of each specimen was assessed to evaluate the remineralization potential of each surface treatment agent. Then, to asses the remineralization potential of various surface treatments at the subsurface level, each enamel specimen was longitudinally sectioned through the centre to expose the subsurface enamel area. Cross-sectional microhardness (CSMH) was evaluated to assess any subsurface remineralization

**Results:**

Statistical analysis using one-way ANOVA followed by multiple comparisons test was applied to detect significant differences at *P* ≤ 0.05 levels between various surface treatments at different phases.

**Conclusions:**

With in the limits, the present study concludes that; ACP-CPP cream is effective, but to a lesser extent than fluoride in remineralizing early enamel caries at surface level. Combination of fluoride and ACP-CPP does not provide any additive remineralization potential compared to fluoride alone. Fluoride, ACP-CPP and their combination are not effective in remineralizing the early enamel caries at the subsurface level.

## INTRODUCTION

A carious lesion begins with the establishment of a combination of specific bacterial population, which is capable of demineralizing enamel under specific modified environment in the oral cavity. This demineralization is clinically manifested as a white, opaque spot particularly when air-dried.

In a neutral environment, the hydroxyapatite of the enamel is in equilibrium with saliva which is saturated with calcium and phosphate ions.[1] At or below pH 5.5, H^+^ ions produced by the bacterial metabolites react preferentially with the phosphate group of the enamel crystals, converting PO4^2−^ ion to (HPO4)^2−^ ion which, once formed, can no more form the crystal lattice; at the same time H^+^ ions are buffered. This leads to enamel dissolution, termed as demineralization, which marks the beginning of early enamel caries.[2,3]

However, the demineralization can be reversed if the pH is neutralized and there are sufficient calcium and phosphate ions available in the immediate environment. This enables the rebuilding of partly dissolved apatite crystals. This is called as remineralization. To restore the natural equilibrium, either remineralization must be enhanced or demineralization must be retarded. The early enamel lesions have a potential for remineralization, with an increased resistance to further acid challenge, particularly with the use of enhanced remineralization treatments.

Fluoride is the most commonly used remineralizing agent. When the acid attacks the enamel surface, the pH begins to rise and fluoride present in the microenvironment causes enamel dissolution to stop.

As the pH rises, new and larger crystals that contain more fluoride (fluorhydroxyapatite) form, thereby, reducing the enamel demineralization by forming fluorhydroxyapatite crystals and enhancing remineralization. Normally, remineralization by fluoride is a self-limiting surface phenomenon that prevents penetration of ions into the depth of the lesion.[4] Rapid deposition of fluorapatite forms a firm surface layer, which is more resistant to further demineralization. However, at the same time, it is resistant to penetration of calcium and phosphate ions required to rebuild the lesion in depth.

A new remineralization technology based on phosphopeptide from milk protein casein has been developed. The casein phosphopeptides (CPP) contain multiphosphoseryl sequences with the ability to stabilize calcium phosphate in nanocomplexes in solutions like amorphous calcium phosphate (ACP). Through their multiple phosphoseryl sequences, CPP binds to ACP in metastable solution preventing the dissolution of calcium and phosphate ions. The ACP-CPP also acts as reservoir of bio-available calcium and phosphate, and maintains the solution supersaturated, thus facilitating remineralization.[5] Studies report that unlike fluoride, ACP-CPP has been shown to remineralize enamel subsurface and subsurface lesion *in vivo* and *in vitro*.[6,7] It is expected that combination of fluoride and ACP-CPP would give enhanced remineralization compared to individual application of fluoride and ACP-CPP. This *in vitro* study aims to evaluate the remineralization potential of fluoride varnish, ACP-CPP, and combination of fluoride +ACP-CPP on early enamel lesions.

## MATERIALS AND METHODS

### The materials used in the study;

1. Fluoride varnish (Fluorprotector Intro pack; Ivaclar Vivadent)
2. Amorphous calcium phosphate- Casein phosphopeptide (CPP-ACP) GC Tooth Mousse, Recaldent; GC Corp; Japan

Fifteen premolars extracted from patients ranging in the age group of 14-20 years, for orthodontic purpose, were collected and the radicular part of each tooth was removed. The coronal part of each tooth was then longitudinally sectioned bucco-lingually and mesio-distally into four sections using a high speed diamond tipped disc. Four enamel specimens were prepared. Custom made plastic cylindrical molds were made and self cured acrylic resin was poured on it; then each enamel block was embedded in, on top of partially set, and allowed to set. An acid resistant nail varnish was applied around the exposed enamel surface leaving a window of 3 mm × 3 mm of enamel exposed at the centre.

Lieca Japan, Tokyo, Vickers micro hardness tester was used to evaluate micro hardness. A load of 25 grams was applied, for five seconds, for all the specimens. The micro hardness numbers (VHN) of five indentations at spacing of 100 microns were taken and the average value was considered the mean base line micro hardness (SMH) of the corresponding specimen. The objective of base line surface micro-hardness determination is to compare and calculate the changes that occur after induction of enamel lesions and after pH cycling.

Carious lesions representing preliminary stage of subsurface enamel demineralization were produced by suspending four sections of each tooth into glass tubes containing 20 ml of demineralization solution, for 72 hours, in an incubator at a temperature of 35 degree.[8] After induction of enamel lesions, all the specimens were evaluated for surface micro hardness measurements under 25 gram loads for five seconds duration.

The composition of the demineralizing solution was as follows;

CaCl_2_ = 2.2 mM NaH_2_ PO_4_ = 2.2 mM Lactic acid = 0.05 M

Fluoride = 0. 2 ppm, Solution was adjusted with 50% NaOH to a pH 4.5

Four sections of each tooth were subjected to the following surface treatments,

Section 1- A thin layer of fluoride varnish was applied, allowed to be absorbed for 20 seconds and then air dried.

Section 2- A generous layer of ACP-CPP cream was applied by an applicator brush and left undisturbed for a minimum of three minutes.

Section 3- A thin layer of fluoride varnish was applied and allowed to be absorbed for 20 seconds. This was followed by a generous layer of ACP-CPP cream and left undisturbed for a minimum of three minutes.

Section 4- This served as the control group where no surface treatment was performed.

A pH cycling regimen included alternative demineralization (three hours) and remineralization (21 hours) for five consecutive days. For the demineralization phase, the demineralization solution used for the induction of enamel lesions was used and for the remineralization phase, a synthetic saliva preparation was carried out.[9]

The inorganic composition of synthetic saliva is similar to that of natural saliva. After pH cycling, again the surface micro hardness was assessed for all the specimens under 25-gram load for 5 seconds.

This composition of the synthetic saliva is as follows:

Na_3_ PO_4_ - 3.90 mM NaCl_2_ - 4.29 mM KCl - 17.98 mM

CaCl_2_ - 1.10 mM MgCl_2_ - 0.08 mM H_2_SO_4_ - 0.50 mM

NaHCO_3_ - 3.27 mM, distilled water, and the pH was set at a level of 7. 2.

Each specimen was longitudinally sectioned into two halves through the center of the window. The cut surface was exposed and polished. A row of five indentations was made at approximately 100 microns below the enamel surface. All the sections were evaluated for the measurement of cross-sectional micro hardness (CSMH) which denoted the changes in micro hardness at subsurface level under the same parameter of load and time. Then the percentage of mineral recovery of the surface micro hardness values was determined by a formula,

% SMHR = Percentage of Surface Micro Hardness Recovery

Initial Enamel IE−Demineralized Enamel DE × 100Treated enamel TE−Demineralized Enamel DE

## RESULTS

Statistical analysis using one-way ANOVA followed by multiple comparisons test (multiple Duncan test)) was applied to detect significant differences at the level of p ≤ 0.05, between various surface treatments at different phases of study.

## DISCUSSIONS

Clinically, the early enamel lesion appears white because the normal translucency of the enamel is lost. The surface becomes fragile and is susceptible to damage from probing. The most important feature of white spot lesion is the presence of relatively intact surface layer overlying subsurface demineralization (40-70%). Even though initial enamel lesions have intact surfaces, they have a low mineral content at the surface layer when compared to sound enamel; thus showing a lower hardness value at the surface than for sound enamel tissue.[10,11]

Organic acids are produced by the metabolic activity of micro organisms in the bacterial plaque. These acids diffuse through the pellicle into the surface enamel. These acids attack the apatite crystals, particularly at the vulnerable lattice points where carbonate ions are present.

This causes Ca^2+^, OH^−^, PO_4_^2−^, F^−^, CO_3_^−^, Na^+^ and Mg^2+^ to be removed from the crystal lattice and to diffuse into the solution phase between the crystals. The dissolving calcium ions and phosphate ions form various calcium phosphate salts that either diffuse to the exterior or provide an environment that facilitates the repair of the faulty crystallites beneath the surface of enamel facilitating remineralization.[12] Mineral loss or demineralization proceeds as long as sufficient acid is available. As more enamel dissolves, concentration of the Ca ion and PO_4_ ion increases.

As calcium and phosphate ions diffuse outwards, remineralization at the surface becomes more and more likely. This leads to the formation of an apparently intact enamel surface layer about 20-40 microns where the mineral content is higher than the body of the lesion.

In the present study, the specimens kept in the demineralization solution (CaCl_2_, NaH_2_ PO_4_, Lactic acid and Fluoride) for 72 hours at 37° C created a subsurface demineralization of approximately 150 microns width with an intact surface simulating an early enamel lesion.[13] The concentration of both calcium and phosphates, in the demineralization solution, was at 50% of saturation level, causing dissolution of only enamel subsurface. Addition of fluoride prevented surface demineralization by forming fluorapatite at the surface, which simulated the naturally occurring early enamel lesions having intact surface layer.

Considering the importance of the surface layer in caries progression, the evaluation of changes in this region is relevant. Surface micro hardness (SMH) measurement is a suitable technique for this purpose. Micro hardness measurement is appropriate for a material having fine microstructure, non-homogenous or prone to cracking like enamel. Surface micro hardness indentation provides a relatively simple, non-destructive and rapid method in demineralization and remineralization studies. Therefore, in the present study, the micro hardness values for each specimen were measured in three steps; the base line micro hardness, after induction of carious lesion (demineralization) and after pH cycling.

The values (VHN) obtained during the initial base line micro hardness measurements in the present study were in the range of VHN 254 – 363, which satisfies the VHN range of normal enamel tissue.[14] The surface micro hardness values for each group of the enamel specimens were decreased to 162-183 at the end of 72 hours of demineralization [Table 1] which is in accordance with the study conducted by Maupome *et al*.[15]

**Table 1:** Comparison of different surface treatment groups at different phases of the study

Observation (SMH)	Fluoride		ACP CPP		Fluoride + ACP CPP		Control	
	Mean	± SD	Mean	± SD	Mean	± SD	Mean	± SD
Initial	313.60	31.36	306.75	23.49	308.75	26.01	299.8	13.56
Demineralization	177.95	17.39	162.85	28.65	168.6	16.93	183.4	19.49
Post pH Cycling	218.30	12.81	185.20	30.79	216.25	16.96	167.3	18.88

The period for demineralization in the pH cycling phase is for three hours, which was to simulate the duration of demineralization (low cariogenic challenge) that occurs in the oral cavity.[16] The test material was applied on enamel blocks twice a day s to simulate the normal recommended daily oral prophylaxis. In the present study, after the pH cycling phase the mean SMH (VHN) for Fluoride group 218.30, for ACP-CPP group 185.20, for Fluoride + ACP-CPP group 216.25 and for the control group 167.30 respectively. It indicates that combination of fluoride + ACP-CPP does not provide any additive remineralization potential when compared to fluoride varnish alone. The mean increase in SMH (VHN) for ACP-CPP treatment group is 185.20, which indicates that there is a significant increase in micro hardness. Therefore, ACP-CPP can also aid in remineralization,[17–21] but not as effectively as fluoride or fluoride and ACP-CPP group combination.

Moreover, in the fluoride + ACP-CPP treatment group, the fluoride varnish was applied first followed by the application of ACP-CPP over the enamel specimens. It is speculated that the results obtained in fluoride + ACP-CPP group reflect the results similar to fluoride varnish and hence might have hindered the effect of ACP-CPP. The varnish applied evaporated quickly to form a thin film on surface. The ACP-CPP group, being creamy in consistency, could not properly wet the surface.

**Graph 1:**
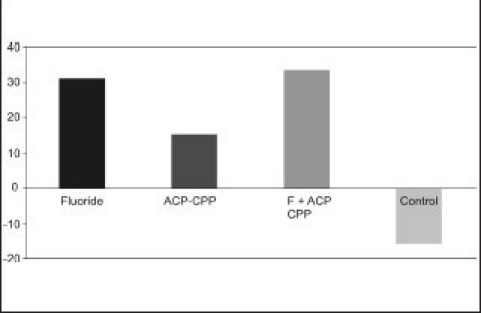
Percentage recovery of surface microhardness

It is speculated that most of the ACP-CPP cream was lost after washing in distilled water. The percentage of surface micro hardness recovery was calculated for all surface treatment groups, which showed greatest recovery for the fluoride + ACP-CPP group (35%) followed by fluoride (32+), followed by ACP-CPP (17+) [Graph 1]. There was no regain in micro hardness in the control group giving a negative sign (−14%). The difference in the percentage micro hardness recovery in fluoride group and fluoride +ACP-CPP group was not statistically significant.

The mean CSMH (VHN) values obtained were: 148.87 (fluoride group), 150.63 (ACP-CPP), 155.51(fluoride + ACP-CPP) and 143.75 (control group) [Table 2]. It indicates that there is no increase in micro hardness at the enamel subsurface, which is not in accordance with the previous studies. There is no remineralization at subsurface level and all the treatment groups failed to remineralize the subsurface lesion in depth. Nevertheless, fluoride, fluoride + ACP-CPP and to a lesser extent ACP-CPP can remineralize the surface lesion. There was no increase in CSMH at the subsurface level and the values suggested that, that none of the surface treatment agents could penetrate the demineralized enamel at the subsurface level. The reason could be; fluoride ions and ACP-CPP were not able to penetrate the subsurface enamel area, the *in vitro* set up is not exactly mimicking the *in vivo* conditions occurring in the mouth, duration of the experimental set up (seven days) is too short.

**Table 2:** Cross-sectional micro hardness comparing different treatment groups after pH cycling

Treatment	Mean	+ SD	F value	*P* value
Fluoride	148.87b	7.55		
ACP CPP	150.63ab	6.78		
Fluoride + ACP CPP	155.51ab	5.64	0.581	> 0.05
Control	145.75b	9.35		

a, b, c: Means with same superscript do not differ each other (Duncan's Multiple Range Test)

## CONCLUSIONS

Within the limits, the present study concludes that; Fluoride varnish is effective in remineralizing the early enamel caries at the surface level. ACP-CPP cream is effective, but to a lesser extent than fluoride varnish in remineralizing early enamel caries at surface level. Combination of fluoride varnish and ACP-CPP does not provide any additive remineralization potential when compared to fluoride varnish alone at the surface level. Fluoride varnish, ACP-CPP cream and combination of fluoride varnish and ACP-CPP are not effective in remineralizing the early enamel caries at the subsurface level. However, one must bear in mind that remineralization *in vitro* may be quite different when compared to dynamic complex biological system which usually occurs in oral cavity *in vivo*. Thus, direct extrapolations to clinical conditions must be exercised with caution because of obvious limitations of *in vitro* studies.
